# Upregulation of myeloid cell leukemia-1 potentially modulates beclin-1-dependent autophagy in ischemic stroke in rats

**DOI:** 10.1186/1471-2202-14-56

**Published:** 2013-05-20

**Authors:** Chen Xingyong, Sun Xicui, Su Huanxing, Ou Jingsong, Huang Yi, Zhang Xu, Huang Ruxun, Pei Zhong

**Affiliations:** 1 Department of Neurology, The First Affiliated Hospital, Sun Yat-Sen University, Guangzhou 510080, PR China; 2 Department of Neurology, Fujian Provincial Hospital, Fujian Medical University, Fuzhou 350001, PR China; 3 State Key Laboratory of Quality Research in Chinese Medicine, Institute of Chinese Medical Sciences, University of Macau, Macao, China; 4 Division of Cardiac Surgery, The First Affiliated Hospital, Sun Yat-sen University, Guangzhou 510080, PR China

**Keywords:** Myeloid cell leukemia 1, Beclin-1, Cerebral ischemia

## Abstract

**Background:**

The mechanisms that underlie autophagy in cerebral ischemia remain poorly defined. Myeloid cell leukemia-1 (Mcl1), an anti-apoptotic member of the Bcl-2 family of proteins, regulates the balance between autophagy and apoptosis. However, little is known regarding its expression profile and contribution to cell fate in the brain following ischemic stroke.

**Results:**

In this study, we investigated the expression profile and cellular distribution of Mcl1 in brains from transient middle cerebral artery occlusion (MCAO) model rats. Brain slices from sham-operated control rats showed minimal immunoreactivity for Mcl1. Mcl1 was mainly produced in neurons. Immunoreactivity for Mcl1 increased as early as 4 hours after MCAO, peaked at 24 hours, and then declined, but still remained high, at 72 hours. Mcl1 positive cells never colocalized with either cleaved caspase-3 or terminal deoxynucleotidyl transferase-mediated dUTP nick-end labeling-positive cells. Both microtubule-associated protein 1 light chain 3 (LC3) and beclin-1 were evident in ischemic brain between 4 and 72 hours after MCAO. Most cells with strong LC3 staining were also labeled with beclin-1. Beclin-1 did colocalize with caspase-3 or Mcl1. Beclin-1/caspase-3 positive cells displayed the characteristic features of apoptosis including cell shrinkage and pyknotic nuclei, whereas beclin-1/Mcl1 positive cells had normal morphology. Pretreatment with 3-methyladenine attenuated autophagy without affecting the level of Mcl1 protein.

**Conclusions:**

These findings demonstrate that the expression of Mcl1 is involved in the survival of neuronal cells. In addition, the coexpression of Mcl1 with beclin-1 may attenuate beclin-1-dependent autophagy during ischemic stroke in rats.

## Background

Autophagy is an intracellular lysosomal degradation process, which is characterized by the formation of double-membraned autophagosomes. Substantial reports have documented that ischemia-induced autophagy leads to neuronal death following ischemic stroke [1,2]. This autophagy-dependent non-apoptotic cell death is defined as autophagic cell death, or type II programmed cell death (PCD), which is characterized by numerous autophagic vacuoles [2,3]. However, the mechanism by which ischemia-induced autophagy promotes cell death remains unclear. Physiologically, autophagy is tightly modulated by regulators to prevent unbalanced activation. Several regulators, particularly the Bcl-2 family of proteins, also have a critical role in the regulation of apoptosis, suggesting that the integrated regulation of autophagy and apoptosis determines cell fate [4]. The Bcl-2 homologue, myeloid cell leukemia 1 (Mcl1), is an anti-apoptotic member of the Bcl-2 family of proteins. Mcl1 is a mitochondrial protein [5] and is believed to inhibit cell death through interactions with pro-apoptotic Bcl-2 family members [6]. Furthermore, Mcl1 also has a key role in regulation of autophagic cell death [6-8]. However, little is known about the expression profile and functions of Mcl1 in the brain following cerebral ischemia.

In this study, we investigated the expression and cellular localization of Mcl1 in the brains of transient middle cerebral artery occlusion (MCAO) model rats. In addition, we examined the potential involvement of Mcl1 in ischemia-induced autophagy following ischemic stroke.

## Results

### Expression of Mcl1 in brain from MCAO rats

**Figure 1:**
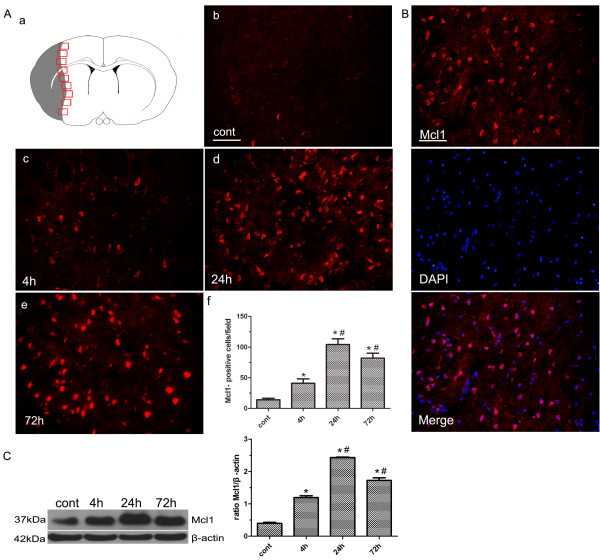
**Expression of Mcl1 in brains from rats subjected to middle cerebral artery occlusion (MCAO).** (**A**) A schematic representation of a coronal brain section. The square fields represent observed regions (**a**). Immunofluorescent staining showed the expression of Mcl1 in brains from sham-operated control (**b**) and MCAO group (4 h, 24 h, 72 h, **c-e**). Quantification of Mcl1-positive cells (**f**). Results were expressed as mean ± SD from four independent experiments. *P < 0.001 vs. control group, ^#^P < 0.001 vs. the previous time point. Scale bars = 50 μm. (**B**) The co-localization of Mcl1 and DAPI indicated Mcl1was mainly localized in the cytoplasm. Scale bars = 50μm. (**C**) Western blot confirmed that Mcl1 protein levels were elevated in MCAO group at each time point compared with control group. Optical densities of respective protein bands were analyzed with Image J 1.42q and normalized to the loading control (β-actin). Results were expressed as mean ± SD from four independent experiments. *P < 0.001 vs. control group, ^#^P < 0.001 vs. the previous time point.

Expression levels of Mcl1 were detected in rats subjected to 1-hour focal ischemia followed by 4, 24 or 72 hours reperfusion. Sparse Mcl1 immunoreactivity was seen in the cortex and striatum of the sham-operated control rats (Figure 1A, b). Mcl1 immunoreactivity started to increase at 4 hours, and further increased at 24 hours. Mcl1 immunoreactivity had declined at 72 hours after 1 hour MCAO, but was still highly expressed (Figure 1A, c-e). Quantification confirmed that the numbers of strongly Mcl1 immunoreactive cells were significantly higher in the ipsilateral ischemic cortex and striatum at all time points compared with the control animals (n = 4/group, ^*^P < 0.001 compared with the control groups, ^#^P < 0.001 compared with the previous time point; Figure 1A, f). At the subcellular level, Mcl1 was mainly localized in the cytoplasm (Figure 1B). Western blot analysis further confirmed that Mcl1 protein levels were elevated in the ipsilateral cerebral cortex and the striatum at all time points when compared with sham-operated control animals (n = 4/group, *P < 0.001 compared with control group, ^#^P < 0.001 compared with the previous time point; Figure 1C). These data indicated that MCAO induced Mcl1 expression in cortical and striatal neurons in a time-dependent manner.

### Cell distribution of Mcl1 in rat brain after MCAO

**Figure 2:**
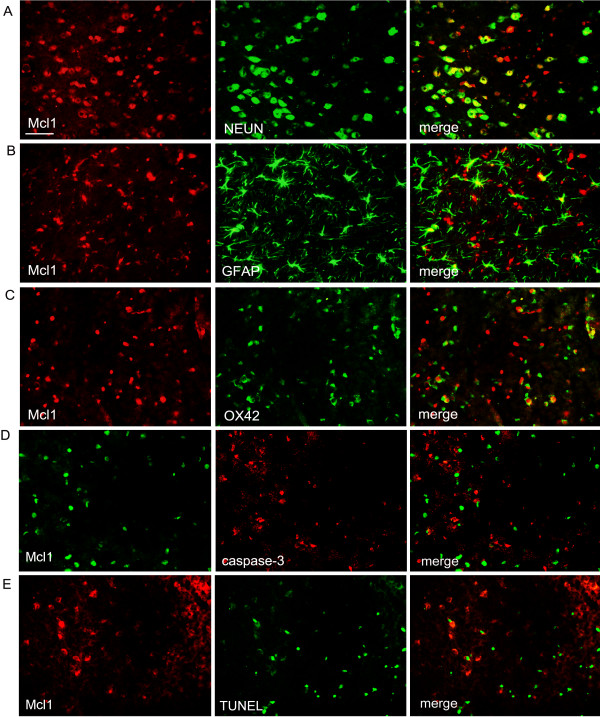
**Cellular distribution of Mcl1 in rat brain after middle cerebral artery occlusion (MCAO).** (**A**) Double immunostaining showed the overwhelming majority of Mcl1 co-localized with NEUN-positive neurons. By contrast, only a few Mcl1 positive cells were labeled with GFAP-positive astrocytes (**B**) or OX42-positive microglia/macrophages (**C**). (**D-E**) Double staining indicated that Mcl1 positive cells did not co-localize with caspase-3 or TUNEL positive cells in the ischemic brain, respectively. Cont: control group. Scale bars = 50 μm.

Cell distribution of Mcl1 expression was further investigated in ischemic brains after MCAO. Antibodies against neuronal nuclei (NEUN), glial fibrillary acidic protein (GFAP) and OX-42 were used to identify neurons, astrocytes and microglia/macrophages, respectively. The overwhelming majority of Mcl1 (red) was labeled with NEUN-positive neurons (green) (Figure 2A). By contrast, only a few Mcl1 positive cells were GFAP-positive astrocytes (green; Figure 2B) or OX42-positive microglia/macrophages (green; Figure 2C), indicating that neurons were the major cell type expressing Mcl1 in the ischemic brain. Double labeling indicated that Mcl1 positive cells did not co-localize with caspase-3 positive cells in the ischemic area (Figure 2D). Furthermore, Mcl1-positive cells were never positive for TUNEL in the ischemic area (Figure 2E), even in the center of the lesion, where TUNEL-positive cells were found at all time points.

### Upregulation of beclin-1 partly contributed to cell death

**Figure 3:**
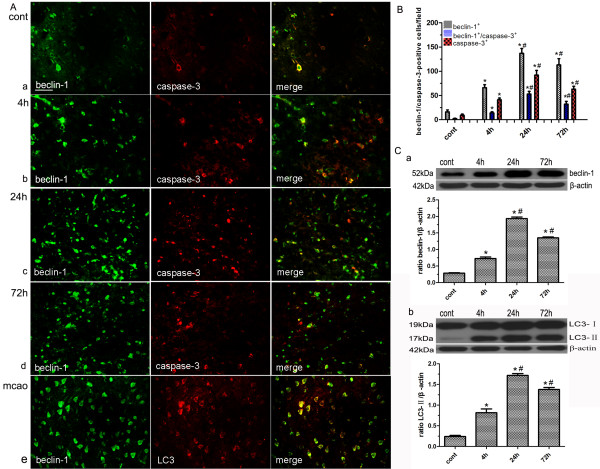
**Upregulation of beclin-1 partly contributed to cell death.** (**A**) Double immunostaining showed the co-localization of beclin-1 and caspase-3 in brains from the control (**a**) and MCAO group (4 h, 24 h, 72 h, **b-d**). Almost all cells with strong LC3 staining were labeled with beclin-1(**e**). (**B**) Quantification of beclin-1^+^, caspase-3^+^ and beclin-1^+^/caspase-3^+^ cells. (**C**) Western blot analysis of protein levels of beclin-1, LC3 and β-actin in the rat cortex and striatum derived from the control and MCAO group. Optical densities of respective protein bands were analyzed with Image J 1.42q and normalized to the loading control (β-actin). Results were expressed as mean ± SD from four independent experiments. *P < 0.001 vs. control group, ^#^P < 0.001 vs. the previous time point. Cont: control group. Scale bars = 50 μm.

Beclin-1 immunoreactivity was weak in brains from sham-operated control rats (Figure 3A, a, left panel). Beclin-1 positive cells increased in the ipsilateral hemisphere in a time-dependent manner after cerebral ischemia. Beclin-1 positive cells started to increase at 4 hours, reaching a peak at 24 hours and declining at 72 hours, but still remained highly expressed after 1 hour of focal cerebral ischemia (Figure 3A, b-d, left panel). Western blotting indicated that beclin-1 protein levels were elevated in the ipsilateral ischemic cerebral cortex and the striatum at all time points when compared with sham-operated control animals (Figure 3C, a). Quantification demonstrated that the numbers of beclin-1 immunoreactive cells and the levels of beclin-1 protein changed from 4 to 72 hours in the ipsilateral ischemic cortex and striatum (n = 4/group, *P < 0.001 compared with control group, ^#^P < 0.001 compared with the previous time point; Figure 3B; C, a). LC3 has been used as a specific marker to monitor autophagy. In the present study, almost all cells with strong punctate LC3 staining were labeled with beclin-1 (Figure 3A, e). A similar expression pattern of LC3 and beclin-1 (Figure 3C), indicated that beclin-1 was a major component in ischemia-induced autophagy.

In order to investigate the relationship between autophagy and apoptosis, brain sections were double-labeled with antibodies against beclin-1 and cleaved caspase-3. The results showed that many beclin-1 expressing cells were negative for cleaved caspase-3, whereas only a small proportion of beclin-1 expressing cells, particularly those located closer to the center of the lesion, were cleaved caspase-3 positive (Figure 3A, a−d). Semi-quantitative assessment showed that among the beclin-1 positive cells, approximately 12.5% of the cells were caspase-3 positive in the sham group, while approximately 21.2% at 4 hours, 38.7% and 28.3% at 24 hours and 72 hours, respectively, were seen in the ipsilateral ischemic hemisphere (n = 4/group, *P < 0.001 compared with control group, ^#^P < 0.001 compared with the previous time point; Figure 3B). Thus, the results suggested that upregulation of beclin-1 was partially implicated in cell death.

### Mcl1 potentially modulated beclin-1-induced autophagy

**Figure 4:**
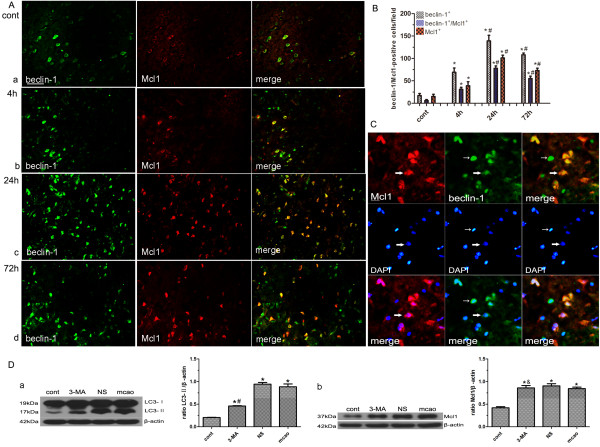
**Mcl1 potentially modulated beclin-1-induced autophagy.** (**A**) Double immunostaining showed the co-localization of beclin-1 and Mcl1 in brains from control (**a**) and MCAO group (4 h, 24 h, 72 h, **b-d**). (**B**) Quantitative assessment the numbers of beclin-1^+^, Mcl1^+^ and beclin-1^+^/caspase-3^+^ cells in control and MCAO group. Results were expressed as mean ± SD from four independent experiments. *P < 0.001 vs. control group, ^#^P < 0.001 vs. the previous time point. (**C**) Some beclin-1expressing cells displayed the characteristic features of apoptosis including cell shrinkage and pyknotic nuclei (small arrow), whereas those cells coexpressing Mcl1 and beclin-1 displayed little chromatin clumping, and their nuclei were not pyknotic, as demonstrated by co-labeling with DAPI (big arrows). (**D**) Pretreatment with 3-MA before MCAO significantly blocked autophagy but did not affect the protein levels of Mcl1. Western blot results showed the levels of LC3-II significantly decreased in 3-MA group compared with that of NS group (**a**). However, Mcl1 protein levels remained unchanged in 3-MA group compared with that of NS group after autophagy inhibition (**b**). Optical densities of respective protein bands were analyzed with Image J 1.42q and normalized to the loading control (β-actin). Results were expressed as mean ± SD from four independent experiments. *P < 0.001 vs. control group; ^#^P < 0.001, ^&^P > 0.10 vs. NS group. Cont: control group. Scale bars = 50 μm (in **A**), 20 μm (in **C**).

Mcl1 is a stress sensor that regulates autophagy and the balance between autophagy and apoptosis [7,8]. Many Mcl1 positive cells also expressed beclin-1 at different time points (Figure 4A, a-d). Semi-quantitative assessment showed that approximately 35.3% beclin-1 positive cells expressed both beclin-1 and Mcl1 in the ipsilateral hemisphere of the control group. Approximately 44.9%, 56.1%, and 50.9% of cells were double labeled in the ipsilateral ischemic hemisphere at 4, 24 and 72 hours, respectively (n = 4/group, *P < 0.001 compared with control group, ^#^P < 0.001 compared with the previous time point; Figure 4B). Results indicated Mcl1 and beclin-1 may operate together in the same cells after ischemic reperfusion. Interestingly, some beclin-1 expressing cells displayed the characteristic features of apoptosis including cell shrinkage and pyknotic nuclei (Figure 4C, small arrow), whereas those cells coexpressing Mcl1 and beclin-1 displayed little chromatin clumping, and their nuclei were not pyknotic as demonstrated by co-labeling with DAPI (Figure 4C, big arrows). The present data suggested that Mcl1 potentially inhibited beclin-1-induced cell death.

To further explore the relationship between Mcl1 and autophagy, rats were pretreated with 3-MA before MCAO. Western blot results indicated that protein expression levels of Mcl1 were not affected after autophagy was blocked by 3-MA pretreatment (Figure 4D, a-b. ^&^P > 0.10, ^#^P < 0.001 compared with NS group; *P < 0.001 compared with control group). The present data suggested that Mcl1 expression may lie upstream of autophagosome formation and potentially modulate beclin-1-induced autophagy.

## Discussion

Mcl1 is a key regulator of apoptosis during central nervous system development and after DNA damage [6,13]. Emerging data suggest that Mcl1 is critical for survival of different cells, whereas inhibition of Mcl1 promotes cell death [14-16]. In the present study, caspase-3 positive cells were not Mcl1 positive, while Mcl1 positive cells were never TUNEL positive at all time points, suggesting that Mcl1 was required for the survival of neural cells following ischemic insult.

Autophagy has been well documented in different models of cerebral ischemia. We consistently found that autophagy, as evidenced by punctate LC3 staining, was significantly increased following focal cerebral ischemia. Furthermore, almost all beclin-1-upregulating cells displayed punctate LC3 fluorescence. This observation indicated that cerebral ischemia may induce autophagy in a beclin-1 dependent-manner. In addition, we found that a subpopulation of beclin-1-positive cells also expressed the active form of caspase-3. These results provided further evidence to demonstrate the close connection between beclin-1-dependent autophagy and cell death [17]. However, not all beclin-1 positive cells were caspase-3 positive, which indicated that not all the beclin-1 positive cells were predestined to die following stroke [18]. Interestingly, some beclin-1 positive cells were also Mcl1 positive, suggesting ischemia-induced coexpression of beclin-1 and Mcl1 in some neural cells. The beclin-1/Mcl1 positive cells had normal morphology and their nuclei were not pyknotic. More importantly, all beclin-1/Mcl1 positive cells were not TUNEL negative. These results suggested that the colocalization of beclin-1 and Mcl1 may favor cell survival following focal cerebral ischemia.

Beclin-1 is essential for the initial steps of autophagy. As one component of the class III phosphatidylinositol kinase (PI3K), beclin-1 initiates autophagy through the interaction with the other components of the PI3K pathway. 3-MA is a relatively selective inhibitor of the class III PI3K and has been shown to inhibit beclin-1-dependent autophagy in different disease models [19]. Pre-administration of 3-MA consistently inhibited the conversion of LC3-I to LC3-II in the ischemic hemisphere following MCAO. However, 3-MA did not affect protein expression of Mcl1, suggesting that PI3K might not be involved in the regulation of the interaction between Mcl1 and beclin-1. One possible explanation is that Mcl1 may interact with beclin-1 to protect against cell death beyond the PI3K complex. Beclin-1 contains a BH3 domain that is sufficient and essential for binding to Bcl-2 homologs [20]. The interaction of Bcl-2 homologs with the BH3 domain can prevent the release of beclin-1, thereby inhibiting beclin-1-dependent autophagy. By contrast, in the absence of Bcl-2 binding, beclin-1 mutants induce excessive autophagy and promote cell death [21]. Indeed, several Bcl-2 family proteins, such as Bcl-2 and Bcl-XL, have been reported to inhibit beclin-1-dependent autophagy through the interaction with the BH3 domain of beclin-1. Given that beclin-1 is localized primarily within cytoplasmic structures, including the endoplasmic reticulum, mitochondria and the perinuclear membrane, and that Mcl1 is a mitochondrial protein [5], it is highly possible that Mcl1 may interact with beclin-1 via the BH3 domain on mitochondria to prevent ischemic cell death [8].

## Conclusions

In summary, the present study demonstrates the expression and cellular localization of Mcl1 in the brains of cerebral ischemia/reperfusion injury model rats. In addition, our findings suggest that expression of Mcl1 is associated with the survival of neurons following ischemic stroke. Mcl1 may inhibit cell death, at least partially through colocalization with beclin-1.

## Methods

### Animals

All experimental procedures were approved by the Institutional Animal Ethical Committee of Sun Yat-sen University and were conducted according to the Guide for the Care and Use of Laboratory Animal of the National Institute of Health (Publication No. 80–23, revised 1996). A total of 60 male Sprague–Dawley rats weighing 250–300 g were purchased from the Center for Experimental Animals of Sun Yat-Sen University. Rats were randomly assigned into four groups: sham-operated control group (cont, n = 10), MCAO group (maco, n = 30), MCAO +3-methyladenine (3-MA) group (3-MA, n = 10), and MCAO + normal saline (NS) group (NS, n = 10).

### MCAO Model

Rats were anesthetized with intraperitoneal (IP) injection of 10% chloral hydrate (3 ml/kg body weight) and subjected to MCAO as described previously, with minor modifications [9]. In brief, a midline neck incision was made, and the right common carotid artery (CCA), external carotid artery (ECA) and internal carotid artery (ICA) were isolated. The ECA was tied. A 4–0 monofilament nylon suture (Beijing Sunbio Biotech Co. Ltd, Beijing, China) with a rounded tip was aseptically inserted from the right CCA to the ICA through the stump of the ECA and gently advanced to occlude the MCA. Recirculation/reperfusion of cerebral blood flow was allowed by gently removing the monofilament after 1-hour ischemia, followed by different time intervals of reperfusion. In sham-operated animals, all procedures except occlusion of the MCA were performed. Core body temperatures were monitored with a rectal probe and maintained at 37°C during the whole procedure. Following surgery, rats were allowed to recover spontaneous breathing and were kept in their cages with free access to food and water. To evaluate impairment of neuronal function after stroke, neurologic examinations were performed 2,4, and 8 hours after the onset of occlusion and then daily until sacrifice by a blinded examiner who used a modified scoring system based on that developed by Longa et al. [9]. The scoring system used is as follows: 0, no deficits; 1, difficulty in fully extending the contralateral forelimb; 2, unable to extend the contralateral forelimb; 3, circling to the contralateral side; 4, falling to the contralateral side; 5, did not walk spontaneously and displayed a depressed level of consciousness.

### 3-Methyladenine administration

One hour before MCAO, rats in the 3-methyladenine (3-MA) group were anaesthetized as above and mounted on a stereotaxic apparatus. 3-Methyladenine (Sigma, St Louis, MO, USA), an autophagy inhibitor, was dissolved in 0.9% saline and injected into the left lateral ventricle at a volume of 10 μL (600 nmol), as previously described [2,10]. Animals in the vehicle group were anaesthetized and injected with the same volume of normal saline. Animals in the sham-operated and MCAO groups did not receive any intraventricular injections.

### Tissue preparation

At 4, 24 and 72 hours after reperfusion, five rats from each group were sacrificed under deep anesthesia with 10% chloral hydrate (5 ml/kg body weight, IP) and then transcardially perfused with 0.9% sodium chloride at 4°C followed by 4% paraformaldehyde in 0.01 M phosphate-buffered saline (PBS, pH 7.4). Brains were then removed, kept in the same fixative for 48 hours at 4°C and cryoprotected in serial PBS isopropanol sucrose solutions (20% and 30%) at 4°C until brains sank. Coronal sections (10 μm) were cut on a cryostat (CM1900; Leica, Heidelberger, Germany) and used for immunofluorescent staining.

### Immunofluorescent labeling

For immunofluorescent assays, frozen sections (10 μm) were prepared using a cryostat (Leica, CM1900) according to standard procedures. The following antibodies were used: rabbit anti-Mcl1 (1:1000; Abcam, Cambridge, UK), mouse anti- Mcl1 (1:100; Santa Cruz Biotechnology, Santa Cruz, CA), mouse anti-NeuN (1:400; Chemicon, Temecula, CA), mouse anti-rat GFAP (1:800; Cell Signaling Technology, Beverly, MA), mouse anti-rat OX-42 (1:300; Millipore, Billerica, MA, USA), mouse anti-rat microtubule-associated protein1 light chain 3 (LC3; 1:100; MBL, Japan), rabbit anti- LC3B (1:2000; Novus Biologicals, USA), and mouse anti-caspase-3 (1:100; Santa Cruz Biotechnology, Santa Cruz, CA). Immunofluorescence was performed as described previously [11,12]. Briefly, sections were pre-incubated with 0.3% Triton X-100 (v/v) in 0.01 M PBS (pH 7.4) for 10 minutes, followed by blocking in 10% normal goat serum (KPL, USA) or 1% bovine serum albumin (MPBIO) for 1 hour at room temperature. Sections were then incubated overnight at 4°C with primary antibodies diluted in primary antibody diluents (Dako, Denmark). After rinsing in 0.01 M PBS (3 × 5 minutes), sections were incubated with FITC-goat anti-rabbit IgG antibodies (1:250; KPL, USA) or Alexa Fluor® 555 conjugated goat anti-rabbit IgG (H + L), F(ab’)2 Fragment (1:1000; Cell Signaling Technology) or Alexa Fluor® 555 conjugated goat anti-mouse IgG (H + L), F(ab’)2 Fragment (1:1000; Cell Signaling Technology) in 0.01M PBS for 1 hour at room temperature. Finally, sections were thoroughly washed (3 × 5 minutes). If necessary, sections were counterstained for nuclei with 4′,6-diamidino-2-phenylindole dihydrochloride (DAPI; 1:1000; Roche, Mannheim, Germany), and then mounted in ProLong® Gold antifade reagent (P36930, Invitrogen) prior to imaging. Fluorescence signal was detected with a microscope (BX51; Olympus). Negative control sections were incubated with PBS instead of primary antibodies and showed no positive staining.

### Western blot experiments

The remaining rats in each group were sacrificed at 4, 24 and 72 hours after reperfusion (five rats in each group). Rats were sacrificed under deep anesthesia with 10% chloral hydrate (5 ml/kg body weight, IP) and then transcardially perfused with 0.9% sodium chloride at 4°C. Brains were then removed, the ipsilateral ischemic cortex and striatum around the infarct area was rapidly dissected from the brain tissue and then homogenized in cell lysis buffer (Cell Signaling Technology, Danvers, MA, USA) with complete protease inhibitor cocktail (Roche). Protein (50 μg) extract from each sample was separated by SDS-PAGE gel electrophoresis (Bio-Rad) and then transferred onto polyvinylidene fluoride membrane (Millipore). Nonspecific binding was blocked with Tris-buffered saline containing 0.1% Tween-20 (TBST) and 5% nonfat milk (MERBCON, BCR685). The membranes were then incubated with primary and secondary antibodies. Primary antibodies used were as follows: rabbit anti-Mcl1 (1:2000; Abcam), rabbit anti-beclin-1 (1:1000; Abcam), rabbit anti-LC3B (1:2000; Novus Biologicals), and mouse monoclonal anti-β-actin (1:3000; Proteintech Group Inc.). Secondary antibodies used were horseradish peroxidase-conjugated goat anti-mouse (1:6000; EarthOx, USA) or goat anti-rabbit IgG antibodies (1:6000; EarthOx). Immunoreactivity was detected with Chemiluminescent HRP Substrate (Millipore) for 5 minutes and then exposed to Kodak X-OMAT films. The exposed X-ray films were scanned. Relative changes in protein expression were estimated from mean pixel density using Image J 1.42q, normalized to β-actin, and calculated as target protein expression/β-actin expression ratios.

### Terminal Deoxynucleotidyl Transferase-Mediated dUTP Nick-End Labeling (TUNEL)

Cell apoptosis was assessed using TUNEL staining. This staining was performed using an in situ cell death detection kit (Roche Applied Science, Nonnenwald, Germany) in accordance with the manufacturer’s instructions. Briefly, brain sections were rinsed three times in PBS, and then were incubated in 0.3% Triton X-100 (v/v) in 0.01 M PBS (pH 7.4) for 20 minutes at room temperature. Subsequently, the TUNEL reaction mixture was then applied for 60 minutes at 37°C. Fluorescence signal was detected using a fluorescence microscope (Olympus BX51) at excitation/emission wavelengths of 492/520 nm (FITC, green).

### Image analysis and quantification

All histological images were analyzed with Image-Pro Plus image analysis software (Media Cybernetics, Silver Spring, MD, USA) by one blinded assessor. The number of immunostaining positive cells was counted using Image-Pro Plus image analysis software in nine comparable, nonoverlapping fields (425 μm × 320 μm; 3 fields per section × 3 sections per rat) under × 400 magnification and was presented as the average cell number per field on each section [11,12].

### Statistical analysis

Data are presented as means ± standard deviation. Statistical analysis was performed by one-way analysis of variance followed by Student’s *t*-test for post hoc analysis. Statistical analysis was performed with SPSS 13.0 for Windows (SPSS Inc., Chicago, IL, USA).

## Abbreviations

Mcl1: Myeloid cell leukemia 1; NS: Normal saline; MCAO: Middle cerebral artery occlusion; CCA: Common carotid artery; ECA: External carotid artery; ICA: Internal carotid artery; 3-MA: 3-methyladenine; DAPI: 4′, 6-diamidino-2-phenylindole dihydrochloride; TUNEL: Terminal deoxynucleotidyl transferase-mediated dUTP nick-end labeling. LC3, microtubule-associated protein 1 light chain 3; IP: Ischemia-reperfusion.

## Competing interests

The authors declare no conflict of interest.

## Authors’ contributions

XC: design of study, in vivo experiments, MCAO model, 3-methyladenine administration, immunofluorescent labeling, tissue preparation, western blot analyses, statistical analyses, first draft of manuscript. ZP: design of study, conception of study, revision of manuscript, final approval of manuscript. RH: design of study, financial support, revision of manuscript, final approval of manuscript. XS and YH: breeding animals. HS, OJ and XZ: revision of manuscript. All authors read and approved the final manuscript.
